# Statement of Retraction: miR-134-5p promotes inflammation and apoptosis of trophoblast cells via regulating FOXP2 transcription in gestational diabetes mellitus

**DOI:** 10.1080/21655979.2024.2299586

**Published:** 2024-02-20

We, the Editors and Publisher of the journal *Bioengineered*, have retracted the following article:

Weiqi Ke, Yixiang Chen, Lijing Zheng, Yuting Zhang, Yudan Wu & Li Li (2022) miR-134-5p promotes inflammation and apoptosis of trophoblast cells via regulating FOXP2 transcription in gestational diabetes mellitus, Bioengineered, 13:1, 319-330, DOI: 10.1080/21655979.2021.2001219

Since publication, significant concerns have been raised about the integrity of the data and reported results in the article. When approached for an explanation, the authors did not provide their original data or any necessary supporting information. As verifying the validity of published work is core to the integrity of the scholarly record, we are therefore retracting the article. All authors listed in this publication has been informed.

We have been informed in our decision-making by our editorial policies and the COPE guidelines.

The retracted article will remain online to maintain the scholarly record, but it will be digitally watermarked on each page as ‘Retracted’.

